# Anterior distal femoral hemiepiphysiodesis can reduce fixed flexion deformity of the knee: a retrospective study of 83 knees

**DOI:** 10.1080/17453674.2018.1485418

**Published:** 2018-06-14

**Authors:** Norbert Stiel, Kornelia Babin, Eik Vettorazzi, Sandra Breyer, Nicola Ebert, Martin Rupprecht, Ralf Stuecker, Alexander S Spiro

**Affiliations:** 1 Department of Pediatric Orthopaedic Surgery, Children’s Hospital Hamburg-Altona, Hamburg, Germany;; 2 Department of Orthopaedics, University Medical Center Hamburg-Eppendorf, Hamburg, Germany;; 3 Department of Medical Biometry and Epidemiology, University Medical Center Hamburg-Eppendorf, Hamburg, Germany

## Abstract

Background and purpose — Fixed knee flexion deformity in children is a common problem in various diseases including myelomeningocele and cerebral palsy. Until now, only a few studies focusing on the surgical procedure of anterior distal femoral hemiepiphysiodesis have been published. We analyzed outcome and correction rate in the largest case series to date of patients treated by staples or 8-plates.

Patients and methods — We reviewed the medical records of all patients with fixed knee flexion deformity who were treated with anterior distal femoral hemiepiphysiodesis using either staples or 8-plates between the years 2002 and 2017 (73 patients; 130 knees). 49 patients (83 knees) had completed treatment with implant removal at the time of full correction of the deformity or at skeletal maturity and were included. The average age at operation was 12 years (6–20). Patients were assigned to 3 different groups based on their diagnosis: cerebral palsy, myelomeningocele, and the “other” group.d

Results — Mean fixed knee flexion deformity improved from 21° (10–60°) to 8° (0–50°) (p < 0.001) with an average correction rate of 0.44° per month (range –2.14° to 1.74°). The correction rate per month was lowest for patients with cerebral palsy (0.20°), followed by the myelomeningocele group (0.50°), and the “other” group (0.58°). Implant loosening occurred in 10% of the treated knees with consecutive re-implantation in 5% of the cases.

Interpretation — Anterior distal femoral hemiepiphysiodesis is an effective and safe method for the treatment of fixed knee flexion deformity in children. The optimal timing depends on the remaining individual growth potential, the underlying disease, and the extent of the deformity.

Fixed knee flexion deformity in children is a common problem in various diseases including arthrogryposis, myelomeningocele, and cerebral palsy (Williams et al. 1993, Wren et al. 2005, van Bosse et al. 2007, van der Krogt et al. 2007). With shortening of muscles with or without spasticity children develop contractures and even bony deformities followed by decreased endurance, knee pain, and progressive crouch gait (Young et al. 2010). Even in non-ambulators knee flexion deformity interferes with activities of daily living (Williams et al. 1993, Murray and Fixsen 1997, Moen et al. 2005, Devalia et al. 2007). Even moderate deformities respond poorly to non-operative treatment like casting, bracing, physical therapy, or local application of botulinum toxin (Molenaers et al. 2006, Westberry et al. 2006, Carbonell et al. 2007). Common surgical procedures include distal femoral extension osteotomy, arthrodiastasis using external fixators, and soft tissue release (Beals 2001, Saraph et al. 2002, Carbonell et al. 2007, Devalia et al. 2007). These procedures are associated with neurovascular risk, infection risk, prolonged postoperative immobilization, fractures, knee instability, and recurrent deformity with continued growth (Devalia et al. 2007, van Bosse et al. 2007, de Morais Filho et al. 2008).

In 2001 Kramer and Stevens published their first data on anterior distal femoral stapling for fixed knee flexion deformity (Kramer and Stevens 2001). Temporary anterior distal femoral hemiepiphysiodesis can be performed by stapling or the implantation of 8-plates. There are few published studies focusing on this surgical procedure. These studies demonstrated an improvement of the deformities combined with a low complication rate due to its less invasive character (Kramer and Stevens 2001, Klatt and Stevens 2008, Palocaren et al. 2010, Macwilliams et al. 2011, Al-Aubaidi et al. 2012). However, none of these studies looked at the correction rates according to the underlying diseases after anterior distal femoral hemiepiphysiodesis.

We reported our early results by using staples and 8-plates in 20 patients in 2012 (Spiro et al. 2012). This is a follow-up study involving all 73 patients who were treated with this procedure at our institution over a 15-year period. We assessed the outcome, complications, and correction rates in relation to the underlying disease or etiology.

## Patients and methods

**Figure 1.**
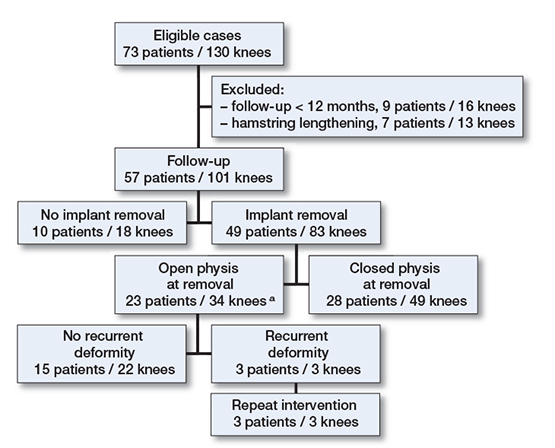
Patients and follow-up. a 4 patients (7 knees) had no follow-up after removal and 1 patient (2 knees) had a permanent epiphysiodesis at the time of implant removal. Some patients had implant removal on one side only, although both sides had been treated.

The medical records of all patients with fixed knee flexion deformity who had been treated with either staples or 8-plates between the years 2002 and 2017 at our institution were reviewed (Figure 1). Inclusion criteria were: (1) fixed knee flexion deformity exceeding 10°, not responding to non-operative treatment (physical therapy, bracing, casting); (2) temporary growth modulation by anterior distal femoral hemiepiphysiodesis; (3) implant removal at the time of full correction of the deformity or at skeletal maturity independent of full correction; and (4) consistent radiographs preoperatively, postoperatively, at the time of implant removal, and during follow-up. Clinical and radiographic follow-up examinations were usually performed every 6 months after surgery. Exclusion criteria were: (1) patients with less than 12 months’ follow-up; (2) dynamic flexion deformities due to spasticity or contracture of the hamstrings; (3) nearly closed distal femoral physis with less than 12 months of predicted growth remaining; and (4) patients who had additional soft-tissue procedures around the knee, such as hamstring lengthening or capsular release.

**Figure 2.**
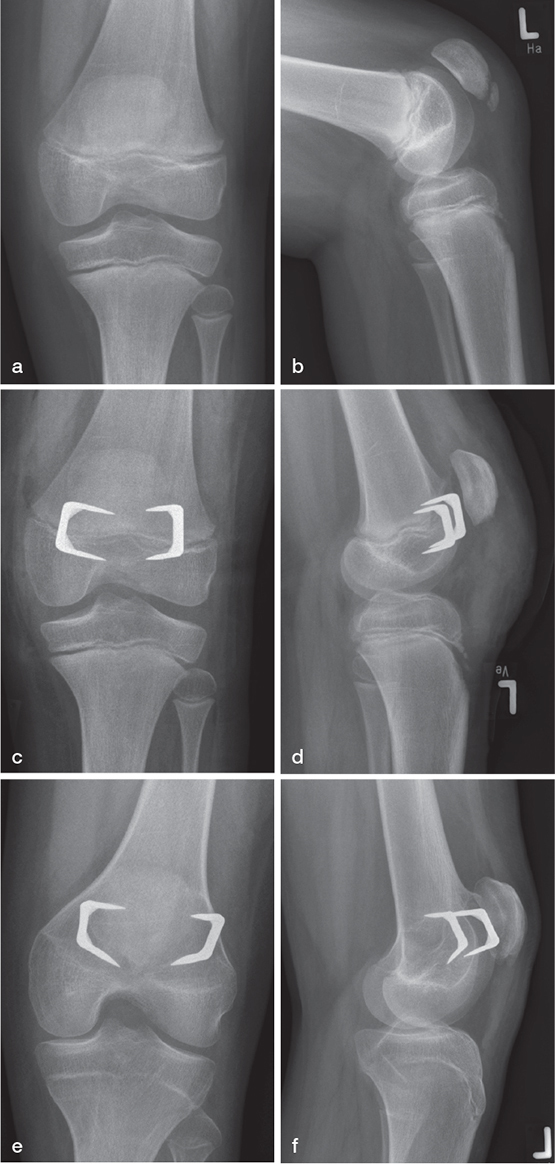
10-year-old boy with myelomeningocele. Before (a, b) and after (c, d) anterior distal femoral hemiepiphysiodesis. Initial radiographs (a, b) show an avulsion fracture of the lower patellar pole in addition. The small patellar fragment was also resected during surgery (c, d). Radiographs taken 3 years after hemiepiphysiodesis and before the implants were removed (e, f). Flexion deformity improved from 10° extension defi ciency to 0°.

The flexion contracture angle, defined as the angle between the neutral position corresponding to 0° and the maximum extension of the knee, was measured with a goniometer. Anteroposterior and lateral radiographs were taken of all knees to document open distal femoral physis preoperatively.

From January 2002 to July 2017, 73 patients (43 males) were treated by anterior distal femoral hemiepiphysiodesis. 49 patients (83 knees) had completed treatment with implant removal at the time of full correction of the deformity or at skeletal maturity and were included. The average age at operation was 12 years (6–20). The mean age was higher in male than in female patients (13.1 vs. 10.5 years; p < 0.001). All patients were under 16 years of age at the time of surgery, except 1 patient who suffered from de Grouchy syndrome. This patient had a chronological age of 20 years, but radiographs showed open distal femoral physis on both sides.

Growth modulation by anterior distal femoral hemiepiphysiodesis was performed on 130 knees (57 bilateral, 16 unilateral fixations). The angle of contracture varied from 10° to 60°. 68 patients were treated with staples and 5 patients with 8-plates. Implants (staples or plates) were removed when full knee extension was achieved or when skeletal maturity had occurred.

### Group assignment

The patients who had completed treatment were assigned to 3 groups according to their diagnosis. Most common diagnoses included cerebral palsy and myelomeningocele (Table 1).

**Table 1.** Group assignment

Etiology of knee flexion deformity	No. of Patients (knees)	Age at surgery years (range)	Age at implant removal years (range)	Time to implant removal months (range)
Cerebral palsy	14 (25)	13 (9–16)	15 (10–21)	38 (6–72)
Myelomeningocele	17 (31)	12 (9–15)	14 (10–18)	31 (12–52)
Other**^**a**^**	18 (27)	12 (6–20)	14 (8–23)	27 (9–63)
Total	49 (83)	12 (6–20)	15 (8–23)	32 (6–72)

**^a^**In the “other” group, 3 patients had a congenital knee flexion deformity, 2 suffered from arthrogryposis multiplex congenita, and 2 developed a contracture after distal femoral fracture. Each of the following diseases occurred once in the “other” group: central core myopathy, de Grouchy syndrome, transverse spinal cord syndrome after embolization, multiple epiphyseal dysplasia, intraspinal lipoma, VACTERL syndrome, congenital knee dislocation, Larsen syndrome, popliteal pterygium syndrome, Omenn syndrome, and skeletal dysplasia.

**Table 2.** Results of hemiepiphysiodesis. Values are degrees

Group	Knee flexion			
	Preop. (range)	At removal (range)	Correction per month (range)	CI
Cerebral palsy	21 (10–50)	11 (0–45)	0.20 (–2.1 to 1.2)	–0.06 to 0.46
Myelomeningocele	20 (10–60)	7 (0–50)	0.52 (–0.30 to 1.7)	0.28 to 0.75
Other **^**a**^**	21 (10–45)	8 (0–45)	0.60 (0 to 1.3)	0.37 to 0.84
Total	21 (10–60)	8 (0–50)	0.44 (–2.1 to 1.7)	

a See Table 1.

### Surgical technique (Figure 2)

All procedures were performed under general anesthesia with the patient supine on a radiolucent table using tourniquets and identification of the distal femoral physis by radiography. Longitudinal incisions of approximately 3 cm were performed on the lateral and medial side of the patella, centered on the physis. An arthrotomy was performed on both sides. After identification of the physis by radiography, a 20-gauge needle was inserted to mark the physis. Thereafter Blount staples were implanted over the physis in a 45° oblique direction. The implanted staples were placed at a distance of 5 mm from the ridge of the femoral sulcus in order to prevent problems with patellofemoral articulation. 8-plates were inserted by using a K-wire instead of a needle for marking the physis. The plates were positioned over the K-wire and fixed using two cannulated screws. Correct position of the implants was verified by fluoroscopy in 2 planes.

After surgery all patients were allowed full weight-bearing and started knee movement immediately as tolerated.

### Statistics

Patient characteristics are reported as mean (range). Due to the presence of dependent parameters taken on both sides (right and left knee), linear mixed models for analysis were used. As the level of significance alpha was set to 0.05. All analyses were performed using R software version 3.3.3 (https://www.r-project.org/).

### Ethics, funding, and potential conflicts of interest

No ethical authorization was required for this type of study. There was no funding. None of the authors have any conflicts of interests or financial disclosures to declare.

## Results (^Table 2^)

All included patients (49 patients; 83 knees) had completed treatment with implant removal at the time of full correction of the deformity or at skeletal maturity. The average follow-up was 46 months (12–78) after implant removal. The average preoperative fixed knee flexion deformity was 21° (range 10°–60°), which improved to 8° (0°–50°). The mean correction of fixed knee flexion deformity was 13° (95% CI (10°–15°), p < 0.001) at the point of implant removal.

Implants were removed after 32 months (6–72) on average, according to a mean correction rate of 0.44° (CI 0.32°–0.60°), p < 0.001) per month. There were no statistically significant differences in the correction of the flexion deformity between males and females (p = 0.1). The highest correction rate per month was found for the patients in the “other” group (0.60°), followed by the myelomeningocele group (0.52°). The lowest correction rate per month was found for the patients with cerebral palsy (0.20°). With regard to the correction rate per month, a difference could be found between the cerebral palsy group and the “other” group. Patients with cerebral palsy had a lower correction rate (p = 0.03). We found a correlation between age at the time of surgery and the degree of correction. The improvement of the flexion deformity decreased with each year of age: delta =1.22° (CI 0.22°–2.23°), p = 0.02.

At the time of implant removal the physis was still open in 23 patients (34 knees). Follow-up examinations to control for rebound deformity were performed for an average of 21 months. 3 patients (3 knees) had a recurrent knee flexion contracture with re-stapling at a time when the distal femoral physis was still open. In 8 of the 49 patients (13 knees) the angle of knee flexion deformity remained unchanged or increased.

1 patient developed a hematoma after staple removal and needed revision surgery. There were no other complications during or after surgeries such as wound infection, neurovascular injury, pathological fractures, or reactive synovitis. Implant loosening occurred in 10% of the treated knees with consecutive re-implantation in 5% of the cases.

## Discussion

In contrast to other surgical procedures, anterior distal femoral hemiepiphysiodesis is a minor operation for fixed knee flexion deformity, it has lower complication risks, and does not require immobilization (Kramer and Stevens 2001). Since Blount and Clarke first mentioned this new method for controlling bone growth by physeal stapling, it has been used successfully to modulate limb-length and correct varus/valgus deformities of the knee in children (Blount and Clarke 1949, Mielke and Stevens 1996, Gorman et al. 2009). The principles of anterior distal femoral hemiepiphysiodesis by using either staples or 8-plates have been described in only a limited number of studies (Kramer and Stevens 2001, Klatt and Stevens 2008, Palocaren et al. 2010, Spiro et al. 2010).

In 2012 we published preliminary data on anterior distal femoral hemiepiphysiodesis. 20 patients with an average age of 13 years were analyzed. We found an improvement in fixed knee flexion deformity from 22° preoperatively to 7° after an average follow-up of 3 years (Spiro et al. 2012). This is a follow-up report with a longer follow-up of the already published data as well as 53 new patients. To our knowledge this is the largest number of patients so far reported in the literature and treated with that procedure. Mean fixed knee flexion deformity improved from 21° (10°–60°) to 8° (10°–50°) with a correction rate of 0.44° per month. Similar results after this procedure have been published by other authors. Klatt and Stevens (2008) found an improvement from 23° to 8° in 23 patients by using 8-plates. Some of their patients had hamstring lengthening in addition. Patients who had fixed knee flexion deformity and hamstring contracture with an increased popliteal angle were treated by anterior distal femoral hemiepiphysiodesis and hamstring lengthening in our study. We excluded these patients and all patients who had capsular release in addition to anterior distal femoral hemiepiphysiodesis in order to avoid any bias. The advantage of anterior distal femoral hemiepiphysiodesis is to avoid complications associated with soft tissue release.

Palocaren et al. (2010) used 8-plates for fixed knee flexion deformity in 10 children (16 knees) with arthrogryposis. Average deformity was 60° preoperatively and was corrected to 33°. Neither screw migration and loosening nor implant breakage was seen in the series of Klatt and Stevens (2008). Palocaren et al. reported only 1 case with plate loosening and consecutive implant removal. Implant loosening occurred in 10% of the treated knees with re-implantation in 5% of the knees in our study. This low proportion of implant loosening after stapling is acceptable. However, in patients with poor bone quality 8-plates for fixation may be preferable.

There have been no detailed analyses of the effectiveness of anterior distal femoral hemiepiphysiodesis focusing on the underlying etiology. The underlying diagnoses had an impact on the correction rate per month when comparing patients with cerebral palsy and patients with “other” diseases. With respect to our 2 largest groups, myelomeningocele and cerebral palsy, the average fixed knee flexion deformity improved from 21° preoperative to 11° in patients with cerebral palsy (correction rate 0.20° per month) and from 20° preoperative to 7° in patients with myelomeningocele (correction rate 0.52° per month). Although patients with myelomeningocele had a higher correction rate than patients with cerebral palsy in our series, the difference was not statistically significant (p = 0.08).

According to patient age at the time of surgery, the improvement of knee flexion deformity decreased by each year of age in this study. Based on these results, we recommend early treatment of fixed knee flexion deformity by anterior distal femoral hemiepiphysiodesis (at around 10 years of age).

Al-Aubaidi et al. (2012) reported one case of stress-related supracondylar femur fracture in a cohort of 8 neuromuscular patients treated with this method. None of our patients developed fractures, neural damage, or deep wound infection. In 3 patients recurrence of deformity was seen requiring repeated correction by hemiepiphysiodesis. In 1 case, after the staple removal, a hematoma developed which needed revision. None of our patients showed impairment of knee flexion or knee stability during the entire follow-up period.

Limitations of our study include its retrospective design. In anterior distal femoral hemiepiphysiodesis the remaining growth potential and therefore the bone age is important. Because bone age was not available for all patients, chronological age was used. Another weakness of the study is the fact that popliteal angle and patella position were not evaluated in each case. Having a large group of patients with long-term follow-up until implant removal is a strength of our study.

In summary, this minimally invasive technique with immediate mobilization and short operation time seems to be an effective treatment for fixed knee flexion deformity in patients with a wide spectrum of underlying diagnoses. We believe that the optimal timing of anterior distal femoral hemiepiphysiodesis especially depends on the remaining individual growth potential, the underlying disease, and the extent of the deformity. A slight overcorrection of fixed knee flexion deformity (about 5 degrees) should be considered in patients with substantial growth potential at the time of implant removal to avoid recurrent knee flexion deformity.

NS and ASS: study design, collection and interpretation of data, and writing. KB, SB, NE, KR and MR: collection of data. EV: statistical analyses, interpretation of data, correction of manuscript. RS: study design and correction of manuscript.

*Acta* thanks Martin Gottliebsen and Niels Wisbech Pedersen for help with peer review of this study.
